# Repeating Task-Irrelevant Contexts Does Not Bias Task Choice and Performance in Voluntary Task Switching

**DOI:** 10.5334/joc.512

**Published:** 2026-08-05

**Authors:** Elena Benini, David Dignath, Moritz Schiltenwolf, Iring Koch, Andrea M. Philipp, Andrea Kiesel

**Affiliations:** 1 Chair of Cognitive and Experimental Psychology, Institute of Psychology, RWTH Aachen University, Germany; 2 Department of Psychology, University of Zurich, Switzerland; 3 Department of Psychology, Eberhard Karls Universität, Tübingen, Germany; 4 Department of Psychology, Ludwig-Maximilians-University, Munich, Germany; 5 Department of Psychology, University of Freiburg, Freiburg, Germany

**Keywords:** Voluntary task switching, feature binding, episodic retrieval, context, adaptive procedure

## Abstract

Goal-directed behaviour requires both efficient task execution as well as adequate task choice. Task choice may reflect deliberate evaluation of costs and benefits, but it may also be biased by bottom-up memory retrieval. Specifically, re-encountering a previously experienced context could retrieve a prior task choice, thereby influencing subsequent decisions. In the present study, using the voluntary task-switching (VTS) paradigm, we examined whether repeating a context would retrieve a task by manipulating whether a task-irrelevant context stimulus repeated or changed across consecutive trials. Based on a feature binding and episodic retrieval framework, we hypothesized that the context—albeit task irrelevant—could become bound with the task in each trial. Consequently, repeating the context would retrieve or reactivate the previous-trial task. Therefore, we predicted that context repetitions would increase task-repetition rates and task-repetition benefits. We used an adaptive VTS procedure (Mittelstädt, Miller, & Kiesel, 2018), wherein the onset of the stimulus affording a task repetition is delayed compared to the stimulus affording a task switch. The delay between the stimuli increases for every consecutive task repetition. We included a task-irrelevant context (a coloured box in Experiments 1 and 2, or a picture in Experiment 3) that could switch or repeat unpredictably in each trial. Contrary to the predictions, we found no evidence that repeating a task-irrelevant context affected either task choice or performance, even when context saliency was enhanced in Experiment 3. We discuss these findings in light of the interplay of performance optimization and task-set availability in VTS.

Humans can flexibly adapt their behaviour to produce certain desired consequences in their environment. For this, they must be able to focus on a certain task and execute it with an appropriate response. Also, they must prevent interference from irrelevant or distracting tasks and environmental stimuli. In addition, to act based on their goals, humans often need to select a task to perform among alternative ones (e.g., should I keep reading this article, or should I write up that discussion section?). The present study examines whether task choice can be influenced by transient associations formed between a task set and a task-irrelevant contextual stimulus. This helps refine our understanding of how goal-directed behaviour is implemented, and the contribution of bottom-up and top-down processes therein.

Multitasking is considered an ideal setting to pinpoint the cognitive control processes underlying goal-directed behaviour, both because managing different tasks at the same time requires control and because it mimics the intrinsically complex human-environment interactions (Hazeltine, 2024; Koch, Poljac, et al., 2018). Different multitasking paradigms were developed to this aim, such as the task-switching paradigm (Rogers & Monsell, 1995; for reviews, see Kiesel et al., 2010; Koch & Kiesel, 2022; Vandierendonck et al., 2010). Here, participants are instructed about two or more tasks and then perform a task in each trial, either based on a pre-established sequence or a task cue. This paradigm is therefore a *forced-choice* task switching. A robust finding is task-repetition benefits, that is, better performance when the task repeats from the previous trial, compared to when it switches. Task-repetition benefits are often referred to as task-switch costs, but the two labels indicate the same measure. Repetition benefits have been attributed to task set reconfiguration processes being necessary in task switches but not in task repetitions (Rogers & Monsell, 1995). However, different theoretical accounts pointed at bottom-up processes being responsible for repetition benefits (Koch & Allport, 2006), such as the carryover of a task representation to the next trial, due to inertia (Allport & Wylie, 1999; Wylie & Allport, 2000).

The voluntary task switching (VTS) paradigm was then developed to obtain a purer measure—compared to forced-choice task switching—of the top-down processes underlying task switching (Arrington & Logan, 2005; Demanet & Liefooghe, 2014). The reasoning is that stimulus-driven bottom-up influences were minimised in VTS, compared to forced-choice task switching, where the task choice is dictated by an external stimulus that requires additional processes such as cue encoding and might trigger cue repetition priming (Schneider & Logan, 2005) and influence performance via task-cue associations (Logan & Bundesen, 2003). In VTS, instead, participants are instructed about two tasks and, in each trial, they decide which task to perform and execute it. Typically, when no instructions about switching behaviour are provided, participants tend to show high repetition rates (80%–90%, Kessler et al., 2009). Since this prevents having enough switch trials to measure task-repetition benefits, often participants are given some randomness instructions, such as choosing the task as if they were flipping a coin in each trial and performing both tasks equally often (Arrington & Logan, 2004). With these instructions, participants usually perform each task equally often, but they choose substantially more task repetitions than switches. Independent of the instructions, participants show task-repetition benefits in VTS as well. This was initially taken as evidence that repetition benefits are apt to measure pure top-down control processes since they occur in a VTS setting where bottom-up influences are minimised.

## Stimulus-Driven Effects on Task Choice

But are bottom-up effects in fact negligible in VTS? To explore the role of bottom-up effects in VTS performance, these studies introduced stimulus repetitions from one trial to the next. Some studies indeed observed larger task-repetition rates with a stimulus repetition (Arrington & Logan, 2005; Mayr & Bell, 2006; see also Demanet et al., 2010, who obtained this result only in a condition of high working-memory load). This indicates bottom-up stimulus-driven influences on task choice (Arrington & Weaver, 2015).

Furthermore, increased stimulus availability seems to affect task choice. This was shown, for example, using distinct stimuli for each task (e.g., a letter for a consonant-vowel task and a digit for an odd-even task), such that a stimulus-onset asynchrony (SOA) can be introduced between the stimuli for each task (e.g., if the stimuli compound is “A7”, one can set the onset of “A” and “7” individually). In this setting, the longer the SOA, the more participants choose the task afforded by the first stimulus, namely the most available (Arrington, 2008). Stimulus availability can also affect repetition rates specifically: Lower repetition rates can be achieved by presenting the stimulus affording a task switch earlier than the stimulus affording a repetition (Mittelstädt, Dignath, et al., 2018).

Furthermore, stimuli might activate the task they were associated with during an earlier training phase. For example, Demanet et al. (2010) observed that, given a stimulus in a test phase, participants were more likely to perform the task that was associated with that stimulus during the training (see also Arrington et al., 2010, who replicated this with only one instance of a given task-stimulus pairing).

Rather than the task itself, a stimulus might retrieve the respective response under a given task (Orr & Weissman, 2011). For example, the stimulus “A” could directly retrieve a “vowel” response instead of retrieving the vowel/consonant task set that, in turn, requires a “vowel” response. However, Orr and Weissman (2011) showed that bottom-up effects on task choice do not exclusively depend on such a stimulus-specific response preparation, but they additionally depend on the availability of each task representation in working memory. This speaks against participants retrieving the response directly without engaging in task selection (e.g., Schneider & Logan, 2015).

Importantly for the current study, task set representations can be strengthened by creating a context that retrieves a task set based on a previously established association. For example, Jurczyk and colleagues (Jurczyk, Mittelstädt, et al., 2021) systematically paired a given foreperiod (long versus short) with each task during a forced-choice task-switching training. In the subsequent VTS test phase, which included the same long versus short foreperiods, participants tended to choose the task previously associated with a given foreperiod.

Taken together, task choice results from a complex interplay of top-down task-set activation (Arrington & Weaver, 2015), as well as bottom-up stimulus-triggered task-set activation.^1^ Note also that stimulus-driven effects are in turn modulated by top-down expectations of the availability of these stimuli themselves (Mittelstädt et al., 2024).

## Contextual Influences on VTS through Binding and Retrieval

In this study, we examined the role of stimulus-driven influences on task choice from the perspective of feature binding and episodic retrieval, specifically within the “binding and retrieval in action control” (BRAC) account (Frings et al., 2020). According to this account, humans bind together elements of each episode, including the action performed, into an event file (Hommel, 2019; Hommel et al., 2001). Such event files are episodic traces that can be retrieved from memory, especially upon repetition of a given element that acts as a retrieval cue. Retrieving an event file causes activation of all the bound features.

Evidence has accumulated for the presence of such binding and retrieval phenomena in experimental settings that require responding to stimuli across consecutive trials (Janczyk et al., 2023). Furthermore, stimuli become bound into event files even if task-irrelevant (e.g., Frings & Rothermund, 2017; Qiu et al., 2022a; Singh et al., 2018), albeit potentially to a lesser extent that depends on the attention deployed on them (i.e., intentional feature weighting; Memelink & Hommel, 2013).

In this study, we examined whether repeating a task-irrelevant context from trial N–1 increased the task-repetition rate in a VTS setting. We derived this hypothesis from the BRAC account, according to which the context would become bound with the task set and the response in each trial. As a consequence, repeating a context can retrieve the N–1 task set, increasing its availability. This study tests this prediction, namely the existence of task set-context binding. Previous studies that investigated this question, albeit within a different theoretical framework, found mixed results. They tested whether repeating the colour of a background box (Arrington & Logan, 2005, Experiments 1–3), a certain shape (Demanet et al., 2010, Experiment 2), or the colour of an irrelevant dots configuration (Jurczyk, Fröber, et al., 2021) in trial N could reactivate the N–1 task set in a bottom-up fashion. Neither Arrington and Logan (2005) nor Jurczyk, Fröber and colleagues (2021) found a greater task-repetition rate when the context repeated, nor did they find any effect on task-repetition benefits. They concluded that the repeating feature must be task-relevant to have an effect (Jurczyk, Fröber, et al., 2021) or that bottom-up processes exert little or no influence on VTS performance (Arrington & Logan, 2005). In contrast, Demanet and colleagues (2010) found a larger task-repetition rate when the context repeated than when it switched (despite the target always switched), suggesting that bottom-up processes might indeed play a role in VTS. Note that repeating the context did not impact task-repetition benefits.

The present study is further motivated by the finding that repeating a task-irrelevant context modulates task switching performance in forced-choice task switching (Benini et al., 2022, 2023, 2024; Schiltenwolf et al., 2024). These studies provide support for the notion that a task-irrelevant context is bound with the task set and the response in each trial and can retrieve them when it repeats in the next trial. Namely, they showed that task-repetition benefits (Schiltenwolf et al., 2024) and/or response-repetition benefits increased when the context repeated compared to switched (Benini et al., 2022, 2023, 2024; see also Dreisbach & Wenke, 2011, who, however, found a modulation of response-repetition costs in task switches). Hence, we aimed to further extend this finding to a setting where such context-triggered retrieval would impact not only task-repetition benefits, but also task-repetition rates.

## The Present Study

According to binding and retrieval accounts (Frings et al., 2020), we hypothesised that the task and the context become bound in each trial, and that repeating the context in the following trial retrieves the bound task. Such an increased availability of the previous-trial task set would then increase task-repetition rates and task-repetition benefits.

The above-described VTS studies investigating whether repeating a context increased repetition rates (Arrington & Logan, 2005; Demanet et al., 2010; Jurczyk, Fröber, et al., 2021) used Arrington and Logan’s (2004) instructions. These instructions consist of the “coin-flip” metaphor (i.e., to choose the task in each trial as if you were flipping a coin), plus the indication of using both tasks equally often. This ensured sufficient task switch trials. More recently, Mittelstädt and colleagues (Mittelstädt, Miller, et al., 2018) devised an adaptive procedure that incentivises task switching without giving instructions about the task sequences. In this study, distinct stimuli served as targets for each task (e.g., a letter stimulus for a vowel/consonant task and a digit stimulus for an odd/even discrimination task). In each trial, the first stimulus to appear (digit vs. letter) is the one that affords a task switch; the stimulus affording a task repetition follows after an SOA. Critically, the SOA increases in given steps (e.g., 50 ms) each time a task repetition is executed. Hence, the more consecutive task repetitions, the longer a participant must wait before being able to perform a task repetition, which eventually leads participants to switch tasks. The instructions emphasize speed and accuracy. Using this procedure, Mittelstädt and colleagues managed to obtain higher switch rates (e.g., 30%–43% across the four experiments of Mittelstädt, Dignath, et al., 2018, and Mittelstädt, Miller, et al., 2018), without giving any instructions about how to choose the task. By de-incentivizing long repetition streaks, this procedure might be blamed for limiting participants’ actual free choice. However, unlike instructions imposing to perform both tasks equally often, this procedure discourages long repetition streaks while in fact leaving participants free to decide the task in each trial.

Using this procedure, Mittelstädt and colleagues reported novel performance-optimization behaviours: Participants with higher repetition benefits are willing to wait longer SOAs before they switch (for the notion of different cost-balancing strategies, see Mittelstädt et al., 2019; Monno et al., 2025). Finally, they replicated the finding that participants with higher repetition benefits tend to switch less (Mayr & Bell, 2006).

### Hypotheses

Using Mittelstädt and colleagues’ adaptive procedure, we examined whether task selection can be influenced by a task-irrelevant context in a procedure that promises to reflect a more genuine—since self-organised—switching behaviour. The same procedure was therefore adopted in all three experiments that only differed in procedural details such as stimulus presentation times and the type and number of contexts used, as detailed below.

The two main preregistered hypotheses for all experiments were (i) higher task-repetition rates when the context repeats than when it switches, due to context-triggered retrieval of the task performed in trial N–1, and (ii) larger task-repetition benefits when the context repeats than when it switches, especially in the reaction times (RTs). To foreshadow the results, however, we did not find evidence for these effects, consistent with Arrington and Logan (2005) and Jurczyk, Fröber, et al.’s (2021) studies.

### Analyses

The main independent variable in the present experiments is context relation (repetition vs. switch), manipulated within-subjects. The dependent variables are task-repetition rate, RTs, and error rates. We examined whether repeating the context would increase the task-repetition rate (dependent variable) by means of a one-tailed paired-sample *t*-test. Furthermore, we examined the effect of repeating the context in combination with repeating the task (an observed within-subjects independent variable) on RTs and error rates (dependent variables) by means of a within-subjects ANOVA.^2^ Furthermore, we ran linear (or logistic, for the errors) mixed models with participants as random intercepts to examine whether two additional within-subjects independent variables, SOA and response repetitions, interacted with task repetition and context repetition and affected RTs and error rates. Note that response repetitions, namely keypress repetitions, were possible only in task repetitions since response keys did not overlap between tasks. By including response repetition as a predictor, we could test whether repeating the context retrieved the previous-trial response, hence benefitting performance in response repetitions.

Additionally, we set out to replicate the positive correlation between task-repetition benefits and task-repetition rate (Mayr & Bell, 2006; Mittelstädt, Miller et al., 2018). This indicates that participants with larger repetition benefits tend to repeat the task more. Furthermore, we computed the average SOA in switch trials for each participant, which represents the average time they were willing to wait (the *waiting SOA*, henceforth). As done in Mittelstädt, Miller et al. (2018), we explored whether the waiting SOA correlated with repetition benefits. A positive correlation indicates that participants with larger repetition benefits accept a longer waiting SOA before switching (Mittelstädt, Miller et al., 2018).

Finally, we conducted non-pre-registered Bayesian *t*-tests to contrast task-repetition rates and task-repetition benefits in context repetitions versus switches. We used a noninformative Jeffreys prior for the variance of the normal population and a Cauchy prior for the standardized effect size with the scaling parameter equal to 0.707. All experiments were preregistered (Exp. 1: https://doi.org/10.23668/psycharchives.12857, Exp 2: https://doi.org/10.23668/psycharchives.13063, Exp 3: https://doi.org/10.23668/psycharchives.14413).

## Experiment 1

### Materials and Methods

#### Power analysis

To derive the required sample size, we relied on the main effect of switching versus repeating the context (i.e., a task-irrelevant coloured box) on task-repetition rate in the study by Arrington & Logan (2005). We calculated the partial eta squared (η_p_^2^) of this effect from the *F* value and the degrees of freedom (as described in Lakens, 2013) in Exp 1 (η_p_^2^ = 0.09), Exp 2 (η_p_^2^ = 0.24), and Exp 3 (η_p_^2^ = 0.07). On average, the effect size was η_p_^2^ = 0.13. We transformed this effect into Cohen’s d for within-subject measures with the formula Cohen’s ${\text{d}}_{\text{z}}\;\text{=}\;\sqrt{\frac{\eta_{p}^{2}}{1\;–\;\eta_{p}^{2}}}$ (Brysbaert, 2019), obtaining d_z_ = 0.39. Using G*Power (Faul et al., 2009), we calculated the sample size needed to detect an effect at least as large as 0.39 with an alpha level of 5%, a power of 80%, and a one-tailed paired-samples *t*-test. Our main hypothesis (H1) indeed consists of a higher task-repetition rate in context repetitions than switches. G*Power returns N = 42 participants to detect such an effect. We rounded it up to the next multiple of eight, namely 48, which allowed us to assign the same number of participants to each of the eight response-key mappings (see Stimuli, Tasks, and Responses).

#### Participants

As per the preregistration, we recruited participants via Prolific who were between 18 and 35 years old, based in Germany, and fluent in English. We collected 49 datasets and we removed three due to participants having more than 90% task-repetition rate, or more than 90% task-switch rate, or fewer than 15 observations per cell, as preregistered (in the preregistration of Experiment 1, we only preregistered to discard participants with more than 90% repetitions, but, consistently with the preregistrations of Experiments 2 and 3, we additionally discarded participants having less than 10% task repetitions as well). Our sample included 11 women and 35 men who were 26.7 years old on average (±4.6 years), and four were left-handed. Participants were compensated with ₤7.54 per hour for an estimated duration of 35 minutes.

#### Stimuli, Tasks, and Responses

**Figure 1:**
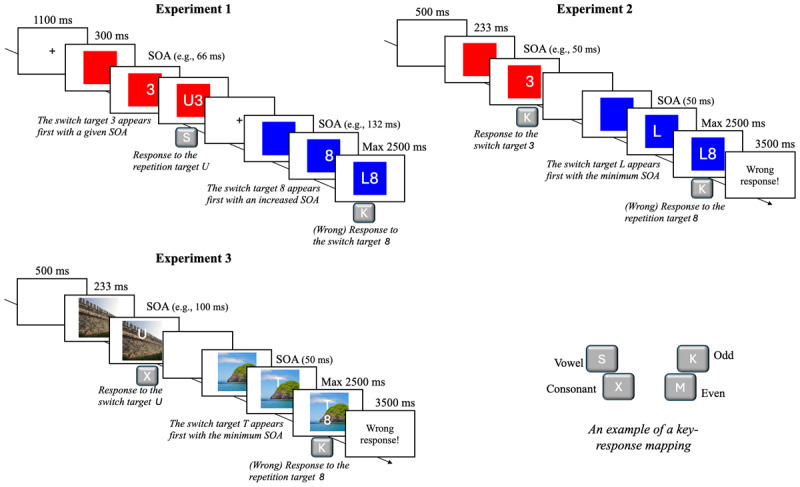
Trials Timelines for all Experiments *Notes*. The pair of trials at the top left illustrates Exp. 1 procedure, where the stimuli were horizontally aligned, the context was either a red or a blue box, the SOA increased in 66 ms steps, and no feedback was provided. In this example, a task repetition (a letter task, in this example) is performed in the first trial. Hence, in the next trial, the SOA increases from, for example, the minimum SOA (66 ms) to the next SOA (132 ms). In this second trial, the example illustrates a wrong response to the switch target (the digit, in this example). The top right trial pair illustrates Exp. 2 procedure, where the stimuli and context were as in Exp. 1, but the SOA increased in 50 ms steps, and feedback was provided for erroneous responses. In this example, a task switch is performed in the first trial, having a hypothetical SOA of 50 ms. Hence, in the next trial, the SOA is reset to the minimum SOA (50 ms). In this second trial, the example illustrates a wrong response to the repetition target. The bottom left trial pair illustrates Exp. 3 procedure, where stimuli were vertically aligned, the context was one of 8 images (only 2 are visible here), and feedback was provided as in Exp. 2. In this example, a task switch is performed in the first trial, having a hypothetical SOA of 100 ms. Hence, in the next trial, the SOA is reset to the minimum SOA (50 ms). In this second trial, the example illustrates a wrong response to the repetition target. The bottom right illustration shows one of the possible key-response mappings, where the vowel/consonant task is performed with the left hand and the parity task with the right hand. Note that, in Exp. 1 and 2, this mapping could only be used when the letter was on the left of the digit (as in this example). When the digit was on the left of the letter, the parity task was performed with the left hand, and the letter task with the right hand.

Context stimuli were a coloured (red or blue) box 200 × 300 pixels presented in the middle of a white background. The targets were two-character strings composed of one digit from 2 to 9 and one letter (A, E, I, U, G, L, N, or R). The string was presented in white at the centre of the context box (see Figure 1). Participants’ tasks were to classify the digit as odd or even, or the letter as a vowel or a consonant. They responded to each task with one hand, pressing the S and × keys with their left middle and index fingers, respectively, and the K and M keys with their right middle and index fingers, respectively. The string was organised to be spatially compatible with the response set. Namely, the digit was always presented to the right of the letter to those participants having the odd/even task mapped onto the right hand. The task-hand assignment as well as the response-key mappings were counterbalanced between participants.

#### Procedure

We programmed the online experiment with Gorilla Experiment Builder (Anwyl-Irvine et al., 2020), where it was also hosted. Data collection took place in May 2023. Participants could only participate using Chrome or Safari as the browser and a computer or laptop as the device.

The first experimental screen reminded participants to pick a quiet place and to find a comfortable position. They were invited to open the study in a new window and/or to enter full-screen mode by pressing the F11 key on the keyboard. They could choose to read the informed consent and the data protection regulation in German or English; after having accepted both, they saw the experiment instructions.

The instructions were in English and explained the tasks and the response-key mapping. The latter were explained both in written form and illustrated with a picture in the instructions showing the hands correctly placed on a keyboard and the response category digitally pasted on each of the four fingers. This picture was shown again before they started the training trials and before each new block of trials. They were asked to respond as fast and accurately as possible, and informed that their response time was calculated from the onset of the first of the two characters. They were also instructed to maintain an overall average accuracy above 55%.

There were 16 training trials where participants saw only a digit per trial and practiced exclusively the odd/even task, followed by 16 trials where they saw only a letter and practised the vowel/consonant task. The order of these single-task training blocks was counterbalanced between participants. Afterwards, they completed 32 training trials that were identical to the experimental trials, hence the VTS procedure described below, except for the presence of accuracy and speed feedback. This was, respectively, a red cross or a green tick presented outside the context box for 400 ms and the sentence: “Please, try to be faster!”, presented if no response was provided before 1500 ms and remaining on the screen till the end of the trial. Upon completing the training, participants could decide to read the instructions again and repeat the training, or start with the experimental trials. The experiment consisted of eight blocks of 64 trials each, so that, in the whole experiment, each of the possible combinations of the eight digits by the eight letters appeared four times with the red context and four times with the blue context. Trial sequences were pseudorandomized to avoid repetitions of either digit or letter from trial N–1.

We employed Mittelstädt, Miller et al.’s (2018) adaptive procedure (see Figure 1). A fixation cross appeared for 1100 ms. Then the context colored-box appeared for 300 ms, after which the first character appeared, followed by the second after a delay equal to the SOA. The first stimulus to appear is the one that, if responded to, causes a task switch; responding to the second stimulus causes a task repetition. The SOA increased by steps of 66 ms each time the same task as in the N–1 trial was performed (the first SOA is 66 ms, the second 132, etc.). The target screen lasted till a response was given or 2500 ms maximum.

During a self-paced break between blocks, participants were shown their overall accuracy and average response time. After completing the eight blocks, participants filled out a demographic questionnaire, and they were thanked and debriefed through a written text explaining the task-context binding hypothesis.

### Results

We analysed the data with RStudio (RStudio Team, 2019), using the package afex (Singmann et al., 2020) for the ANOVA and mixed models, and with the BayesFactor (Morey & Rouder, 2024) package for calculating Bayesian *t*-tests. Importantly, RTs are calculated from the onset of the respective stimulus; hence, the SOA is subtracted from the RTs in repetition trials (note that participants were instead prompted to respond as fast as possible, considering the SOA). We cleaned the data as per the preregistration, so for each participant, we computed their average RT and removed those trials slower than three standard deviations from the average. We also removed trials faster than 200 ms, the first trial of each block (which cannot be classified as a switch nor a repetition), the trials where no response was given before the deadline (i.e., the time-outs), trials where a response was given before the respective stimulus onset, and the trials following an error. In the analyses of the RTs (with task relation and context relation as predictors) and of the task-repetition rate (with context relation as the predictor), but not in the ERs analysis (with task relation and context relation as predictors), we also removed error trials. For the RTs, we analysed 85.4% of the raw data and 90.5% for the errors.

#### Context Repetitions Effects on Task-Repetition Rate and Task-Repetition Benefits

**Figure 2:**
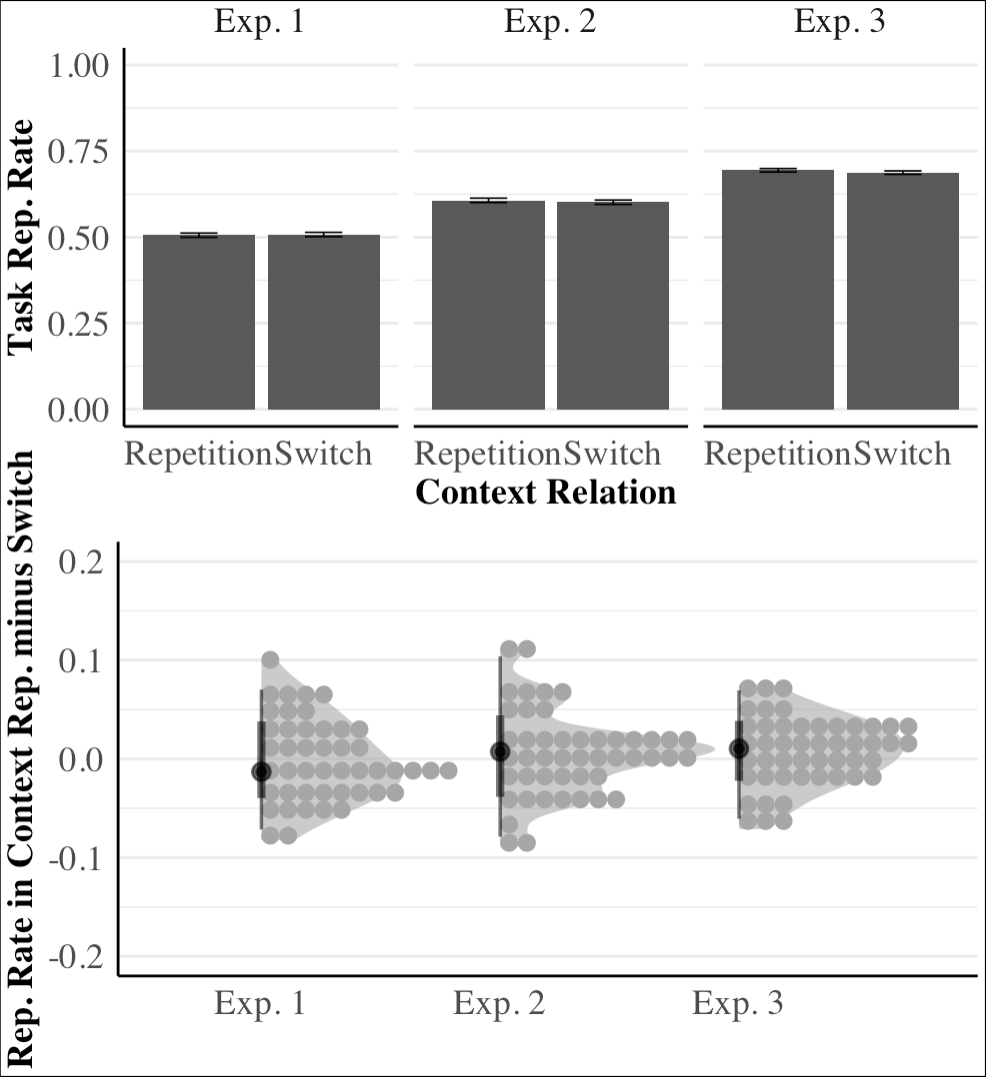
Mean Task-repetition rate based on Context Relation in all Experiments *Notes*. Rep. = Repetition. The upper panel depicts the mean task-repetition rate in context repetitions versus switches in the three experiments. Vertical bars represent 95% confidence intervals of the difference of the means. The lower panel depicts violin plots as well as individual participants’ means of the effect of interest, namely task-repetition rates in context repetitions minus task-repetition rates in context switches.

**Figure 3:**
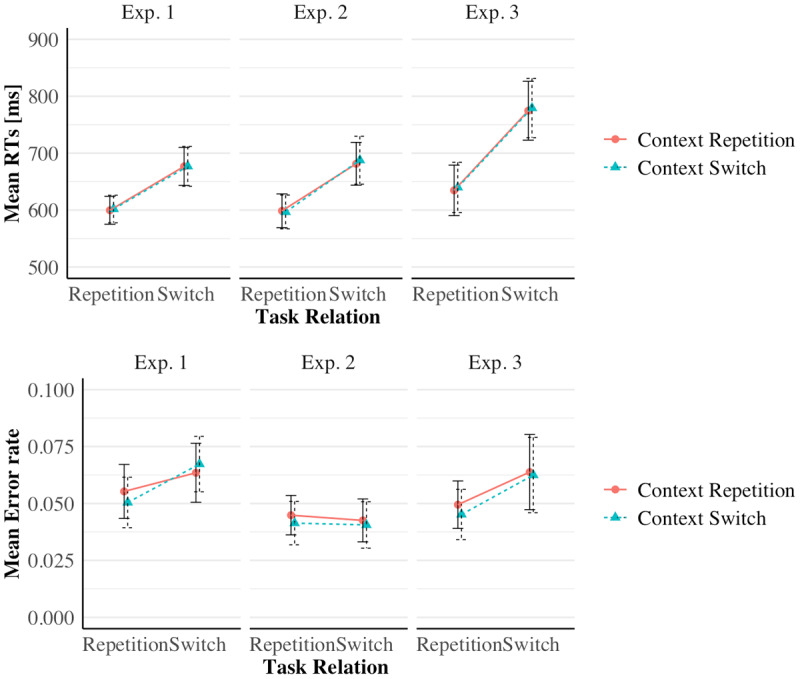
Mean RTs and Error Rates based on Context Relation in all Experiments. *Notes*. The vertical bars represent 95% confidence intervals of each raw mean.

The results are illustrated in Figures 2 and 3, left panels. Task-repetition rates did not differ between context repetitions and switches (49.8% vs. 50.2%, in context repetitions vs. switches), *t*(45) = –0.60, *p* = .724, *d_z_* = –0.09. The Bayesian *t*-test indicated substantial evidence for the absence of an effect, BF_01_ = 5.3.

In the ANOVA on the RTs, we found a significant main effect of task relation, *F*(1, 45) = 54.17, *p* < .001, η_p_^2^ = 0.55, η_G_^2^ = 0.115, indicating 74 ms task-repetition benefits. The main effect of context was not significant, *F*(1, 45) = 0.99, *p* = .325, η_p_^2^ = .02, η_G_^2^ < .001, nor was the interaction of task relation and context relation, *F*(1, 45) = 0.62, *p* = .437, η_p_^2^ = .01, η_G_^2^ < .001.

In the ANOVA on the error rates, we found a significant main effect of task relation, *F*(1, 45) = 6.58, *p* = .014, η_p_^2^ = .13, η_G_^2^ = .023, indicating 1.2% task-repetition benefits. The main effect of context was not significant, *F*(1, 45) = 0.03, *p* = .872, η_p_^2^ < .001, η_G_^2^ < .001, nor was the interaction of task relation and context relation, *F*(1, 45) = 1.72, *p* = .196, η_p_^2^ = .04, η_G_^2^ = .003. The Bayesian *t*-tests on task repetition benefits in context repetitions versus switches indicated substantial evidence for the null hypothesis for RTs, BF_01_ = 5.6, but only inconclusive evidence for the null for errors, BF_01_ = 2.8.

#### SOA and Response-Repetition Effects on RTs and Errors

The results of the mixed models on RTs and error probability, including task relation, context relation, response relation, and SOA, are reported in Table 1. Despite response repetitions (i.e., same keypress in subsequent trials) being only possible in task repetitions, this model also includes task switches to examine how task and context relation interacted with SOA. For brevity, we only discuss the significant results of SOA and response relation and omit the effects of task relation and context relation that we discussed above.

**Table 1:** Mixed Models on Reaction Times and Errors of Experiment 1.


FIXED EFFECT	REACTION TIMES			ERROR			
	
	ESTIMATE	*t* value	*p*	ESTIMATE	*z* value	*p*	ODD RATIO

(Intercept)	632.019	46.889	<.001	–2.798	–26.865	<.001	0.061

Task Rep.	22.161	9.625	<.001	0.265	6.3	<.001	1.304

Context Rep.	0.979	0.422	0.673	0.019	0.521	0.602	1.019

SOA	0.034	3.71	<.001	0.074	2.111	<.05	1.077

Response Rep.	–2.053	–0.811	0.417	–0.303	–6.572	<.001	0.738

Task Rep.:Context Rep.	1.171	0.545	0.586	0.05	1.225	0.22	1.051

Task Rep.:SOA	0.083	9.726	<.001	0.109	2.682	<.01	1.115

Context Rep.:SOA	0.014	1.635	0.102	0.004	0.116	0.908	1.004

Context Rep.:Response Rep.	–6.072	–2.403	0.016	–0.027	–0.593	0.553	0.973

SOA:Response Rep.	–0.001	–0.114	0.909	–0.075	–2.03	<.05	0.928

Task Rep.:Context Rep.:SOA	0.003	0.396	0.692	–0.047	–1.182	0.237	0.954

Context Rep.:SOA:Response Rep.	0.006	0.797	0.425	0.023	0.62	0.536	1.023


*Note*. Rep. = Repetition. The variables are sum-coded such that Switch = 1 and Repetition = –1. SOA values were standardized. The model formula was Task_rep*Context_rep*SOA + Response_rep*Context_rep*SOA + (1|pp).

In the model predicting RTs, there was a significant main effect of SOA, indicating that RTs increased with longer SOAs. This effect was further qualified by an interaction with task relation, indicating that task-repetition benefits increased with longer SOAs. We found a two-way interaction of response relation and context relation, indicating larger response-repetition benefits in context repetitions (36 ms benefits) than in context switches (19 ms benefits).

In the generalised mixed model on the probability of making an error, we found a significant main effect of task relation and of response relation, indicating both task and response-repetition benefits. There was a significant main effect of SOA, indicating a larger probability of error with longer SOA, consistent with the RTs model. This effect interacted with task relation, indicating that task-repetition benefits increased with longer SOA, as in the RTs. Finally, we found a main effect of response relation, indicating that the probability of making an error was approximately 30% more in response repetitions than in switches. This interacted with SOA, indicating that these response-repetition costs diminished with longer SOAs.

#### Correlations between Repetition Benefits and Switching Behaviour

Consistent with previous studies, the task-repetition rate was positively correlated with task-repetition benefits, ρ = 0.39, *t*(44) = 2.82, *p* = .007, indicating that participants with higher repetition benefits on average were also more willing to repeat the task. Furthermore, task-repetition benefits also positively correlated with the waiting SOA, ρ = 0.30, *t*(44) = 2.07, *p* = .044, indicating that participants with higher repetition benefits on average were also willing to wait longer SOAs before switching the task. However, participants’ average SOA in switch trials (175 ms) was significantly larger than their average repetition benefit (73 ms), *t*(45) = 7.87, *p* < .001, *d_z_* = 1.2, indicating they were willing to wait a SOA longer than their repetition benefit before they switched, namely a suboptimal willingness to wait.

### Discussion

We hypothesized that repeating the context may retrieve the N–1 task set if the task set and context became bound in trial N–1. Hence, we predicted a larger probability of repeating the task (H1) and larger task-repetition benefits when the context repeated compared to switched. The results of Experiment 1 showed neither of the predicted effects, with the Bayesian Factors providing substantial evidence for the absence of these effects, except for the error rates, where this evidence was only anecdotal. The null results might be due to no binding in trial N–1, as well as no retrieval in trial N, or both.

The mixed model on RTs provided some evidence for context repetition impacting performance in a way consistent with binding and retrieval, as context repetitions sped up response repetitions. Since response repetitions were only possible in task repetitions, this indicates that context effects emerged especially in task repetitions. This finding is consistent with the context being bound with the N–1 *response* and retrieving it when the context repeats, improving response-repetition performance. This also aligns with the findings of Benini et al. (2022, 2023, 2024) and Koch, Frings, et al. (2018).

Note, however, that response repetitions were less accurate than response switches. Although this was not modulated by context relation in the errors model, it indicates that the faster response repetitions when the context repeated did not necessarily reflect improved performance, and might instead suggest a speed-accuracy trade-off.

Furthermore, the results of both models indicate that longer SOAs increased task-repetition benefits. We observed this pattern in Experiments 2 and 3 as well; hence, we return to and elaborate on this result in the General Discussion.

Finally, we replicated the positive correlations between task-repetition benefits and both task-repetition rate and the waiting SOA, indicating that participants with larger repetition benefits preferred to switch less and waited a longer SOA before switching. However, the average waiting SOA was larger than the average repetition benefits, indicating a suboptimal organisation of participants’ performance.

## Experiment 2

Experiment 2 was aimed at replicating Experiment 1 in an independent dataset while applying minor changes to the procedure. Specifically, we reduced the SOA steps to obtain a more fine-grained assessment of participants’ switching behaviour in relation to the SOA (e.g., Monno et al., 2021), and we introduced a long error feedback screen, appearing after each error, which should incentivise accuracy.

### Materials and Methods

The power analyses, the sample inclusion criteria, the tasks and stimuli, and most of the procedure were as in Experiment 1. In what follows, we only report what deviated from Experiment 1.

#### Participants

We collected 48 datasets, but we discarded one with fewer than 15 observations in the task switch with context switch design cell. Hence, we analysed 47 datasets (15 females, 1 diverse, and 31 males) wherein the average age was 26.7 years (±4.3 years), and five were left-handed. Participants were compensated with ₤7.5 per hour for an estimated duration of 30 minutes.

#### Stimuli, Tasks, and Responses

Stimuli, tasks, and responses were as in Experiment 1.

#### Procedure

Data collection took place in August 2023. The procedure of Experiment 2 was substantially the same as Experiment 1 except for the differences described here (see also Figure 1). The SOA increased by steps of 50 ms instead of 66 ms. The fixation cross presented between the response in trial N–1 and the onset of the context was presented for 500 ms instead of 1100 ms, and the context appeared 250 ms (and not 300 ms) before the first stimulus. Finally, after an error, participants saw an error-feedback screen recapping the response key mapping for 3.5 seconds, while there was no error feedback in the experimental trials of Experiment 1.

### Results

We cleaned and analysed the data as in Experiment 1. After data cleaning, we analysed 88.4% of the raw data for the RT analyses, and 92.3% for the errors analyses.

#### Context Repetitions Effects on Task-Repetition Rate and Task-Repetition Benefits

The results are visible in Figures 2 and 3, middle panels. Task-repetition rates were not higher in context repetitions than switches (60.2% vs. 60.6%, in context repetitions vs. switches), *t*(46) = 0.69, *p* = .247, *d_z_* = 0.10. The Bayesian *t*-test indicated substantial evidence for the absence of an effect, BF_01_ = 5.0.

In the ANOVA on the RTs, we found a significant main effect of task relation, *F*(1, 46) = 65.86, *p* < .001, η1_p_^2^ = 0.59, η_G_^2^ = 0.11, indicating 84 ms task-repetition benefits. The main effect of context was not significant, *F*(1, 46) = 0.12, *p* = .728, η_p_^2^ = .003, η_G_^2^ < .001, nor was the interaction of task relation and context relation, *F*(1, 46) = 0.71, *p* = .403, η_p_^2^ = .015, η_G_^2^ < .001.

In the ANOVA on the error rates, we surprisingly did not find significant task-repetition benefits (1.5%), *F*(1, 46) = 0.17, *p* = .682, η_p_^2^ = .004, η_G_^2^ < .001. The main effect of context was not significant, *F*(1, 46) = 0.49, *p* = .486, η_p_^2^ = .011, η_G_^2^ = .002, nor was the interaction of task relation and context relation *F*(1, 46) = 0.05, *p* = .830, η_p_^2^ = .001, η_G_^2^ < 0.001. Both Bayesian *t*-tests on task repetition benefits in context repetitions versus switches indicated substantial evidence for the null hypothesis (for RTs, BF_01_ = 4.5; for errors, BF_01_ = 6.2).

#### SOA and Response-Repetition Effects on RTs and Error Rates

The results of the mixed models on RTs and error probability are reported in Table 2. For brevity, we only discuss the significant results of SOA and response relation.

**Table 2:** Mixed Models on Reaction Times and Errors of Experiment 2.


FIXED EFFECT	REACTION TIMES			ERROR			
	
	ESTIMATE	*t* value	*p*	ESTIMATE	*z* value	*p*	ODD RATIO

(Intercept)	625.625	39.042	<.001	–3.126	–37.272	<.001	0.044

Task Rep.	21.538	10.259	<.001	0.081	1.775	0.076	1.084

Context Rep.	0.569	0.279	0.78	–0.042	–0.987	0.324	0.959

SOA	0.051	5.676	<.001	–0.006	–0.105	0.917	0.994

Response Rep.	6.396	3.061	0.002	–0.216	–4.915	<.001	0.806

Task Rep.:Context Rep.	0.929	0.489	0.625	0.026	0.589	0.556	1.026

Task Rep.:SOA	0.104	12.206	<.001	–0.058	–1.071	0.284	0.944

Context Rep.:SOA	0.009	1.052	0.293	–0.062	–1.194	0.232	0.94

Context Rep.:Response Rep.	–3.397	–1.628	0.103	0.003	0.066	0.947	1.003

SOA:Response Rep.	–0.011	–1.695	0.09	0.021	0.561	0.575	1.021

Task Rep.:Context Rep.:SOA	0.01	1.174	0.24	–0.035	–0.67	0.503	0.966

Context Rep.:SOA:Response Rep.	0.005	0.81	0.418	–0.006	–0.161	0.872	0.994


*Note*. Rep. = Repetition. The variables are sum-coded such that Switch = 1 and Repetition = –1. SOA values were standardized. The model formula was Task_rep*Context_rep*SOA + Response_rep*Context_rep*SOA + (1|pp), and it was fitted with the bobyqa optimizer as opposed to the default, which did not converge (nloptwrap).

In the linear mixed model on RTs, as in Experiment 1, there was a significant main effect of SOA, indicating that RTs increased with longer SOAs. This effect was further qualified by an interaction with task relation, indicating that task-repetition benefits increase with longer SOAs. We found a main effect of response relation, indicating longer RTs in response switches (636 ms) than in repetitions (595 ms), and the interaction with context relation was not significant.

In the generalised mixed model predicting the probability of making an error, we found a main effect of response relation indicating a higher probability of making an error in response repetitions (5.1%) than switches (3.9%), as in Experiment 1.

#### Correlations between Repetition Benefits and Switching Behaviour

As in Experiment 1, task-repetition rate was positively correlated with task-repetition benefits, ρ = 0.48, *t*(45) = 3.69, *p* < .001, indicating that participants with higher repetition benefits on average were also more willing to repeat the task. Larger repetition benefits also implied longer waiting SOA, ρ = 0.45, *t*(45) = 3.43, *p* = .001, indicating that participants with higher repetition benefits on average were also willing to wait longer SOAs before switching the task. However, as in Experiment 1, participants’ average waiting SOA (170 ms) was significantly larger than their average repetition benefit (85 ms), *t*(46) = 7.50, *p* < .001, *d_z_* = 1.1, indicating an excessive willingness to wait compared to their individual average repetition benefits.

### Discussion

The results of Experiment 2 mostly replicated Experiment 1. Our main hypotheses were not confirmed since context repetitions did not increase task-repetition rates nor task-repetition benefits. The absence of an effect was supported by all the Bayesian t-tests, providing substantial evidence for the null. Different from Experiment 1, context repetition did not speed up response repetitions either, which, in Experiment 1, we discussed as consistent with context-response binding. In this experiment, we obtained a clear indication of a speed-accuracy trade-off involving response repetitions, which were both faster and less accurate than switches (in Experiment 1, response repetitions were less accurate but not faster). As in Experiment 1, longer SOA increased task-repetition benefits. Finally, participants with larger repetition benefits showed greater repetition rates and longer waiting SOA. However, the average waiting SOA was larger than the average repetition benefits, as in Experiment 1.

## Experiment 3

In Experiment 3, we attempted to strengthen the effect of context that we predicted but failed to observe in Experiments 1 and 2. To this aim, we increased context saliency by using colourful pictures of objects or landscapes. Furthermore, we used eight different context pictures as opposed to the two coloured boxes of the previous experiments. Increasing context variability should increase the saliency of the context itself, hence increasing the probability that this enters event files (Qiu et al., 2022a, 2022b; Qiu & Mo, 2024) as well as increasing the distinctiveness of a context repetition, possibly strengthening context-triggered retrieval of the bound event-file.

### Materials and Methods

The power analyses, the sample inclusion criteria, and the tasks were as in Experiment 2. All the differences are reported below.

#### Participants

We collected 48 datasets (15 females, 2 diverse, and 31 males) wherein the average age was 26.5 years (±4.4 years), and five were left-handed. Participants were compensated with ₤7.5 per hour for an estimated duration of 30 minutes.

#### Stimuli, Tasks, and Responses

The stimuli, tasks, and responses were as in Experiments 1 and 2. The characters were, however, arranged vertically, instead of horizontally, such that the onset of a stimulus would not prime the ipsilateral hand (see Figure 1). We counterbalanced between participants whether the digit was presented above or below the letter. As a consequence of this vertical stimulus organisation, stimulus positions did not constrain task-hand mapping anymore, so both assignments of a task to a hand were possible, and were counterbalanced between participants. The context was not a blue or a red box, but rather a 512 × 512 pixel picture presented underneath the characters, in the same position as in the previous two experiments. The picture was one of eight AI-generated pictures representing different scenarios (a beach, a room, a forest, a boat, etc.). The pictures are available together with the data and analysis scripts of all the experiments at: https://doi.org/10.23668/psycharchives.21595.

#### Procedure

The procedure of Experiment 3 was the same as in Experiment 2, but trial sequences were pseudorandomized differently. We again avoided N–1 repetitions of either letter or digit, but we did so without biasing the probability that a certain stimulus category would switch. In the sequences of Experiments 1 and 2, avoiding N–1 stimulus repetitions rendered the probability that both stimulus categories repeated lower than the probability that neither repeated. For example, a double switch such as odd → even and vowel → consonant was approximately 1.5 times more likely than a double repetition such as odd → odd and vowel → vowel. In Experiment 3, we instead equalized the probability of these two transitions. This means that pressing the same key in subsequent trials (i.e., a task repetition with a response repetition) would yield a correct response in 50% of the trials.

As in the previous experiments, each of the 64 combinations of digits and letters was presented 8 times in the whole experiment. The assignment of a context picture to each combination was instead randomised, hence different for each participant, and due to the randomness, a certain picture might have appeared more frequently with a certain letter or digit. Despite having eight and not two contexts, we kept context repetitions to 50% as in the previous experiments, with the following algorithm: A sequence with 50% “repetitions” and “switches” is generated, then a random context is extracted to be presented in trial one. For the following trials, if the trial was a repetition, then the same context was presented; if it was a switch, one of the other seven pictures was drawn, with the constraint that the same picture was not already presented in the seven preceding trials. Compared to random assignment, this increased the distance between two occurrences of a context image, which should augment the saliency of a context repetition, as well as increasing the probability that each context picture was used a similar number of times in the experiment.

### Results

We cleaned and analysed the data as in Experiments 1 and 2. After data cleaning, we analysed 86.0% of the raw data for the RT analyses, and 90.6% for the errors analyses.

#### Context Repetitions Effects on Task-Repetition Rate and Task-Repetition Benefits

The results are presented in Figures 2 and 3, right panels. Task-repetition rates were not higher in context repetitions than switches (69.2% vs. 69.8%, in context repetitions vs. switches), *t*(47) = 1.34, *p* = .094, *d_z_* = 0.19. The Bayesian *t*-test indicated inconclusive evidence for the absence of an effect, BF_01_ = 2.8.

In the ANOVA on the RTs, we found a significant main effect of task relation, *F*(1, 47) = 85.83, *p* < .001, η_p_^2^ = .646, η_G_^2^ = .135, indicating large task-repetition benefits (136 ms). Different from the previous experiments, the main effect of context was significant, *F*(1, 47) = 4.70, *p* = .035, η_p_^2^ = .091, η_G_^2^ < .001, indicating context-repetition benefits (9 ms), but context did not interact with task relation, *F*(1, 47) = 0.52, *p* = .475, η_p_^2^ = .011, η_G_^2^ < .001.

In the ANOVA on the error rates, we found a significant main effect of task relation that we did not find in the error rates of Experiment 2, *F*(1, 47) = 13.96, *p* < .001, η_p_^2^ = .229, η_G_^2^ = .026, indicating 1.6% task-repetition benefits. The main effect of context was not significant, *F*(1, 47) = 0.81, *p* = .372, η_p_^2^ = .017, η_G_^2^ < .001, nor was the interaction of task relation and context relation, *F*(1, 47) = 0.15, *p* = .703, η_p_^2^ = .003, η_G_^2^ < .001. Both Bayesian *t*-tests on task repetition benefits in context repetitions versus switches indicated substantial evidence for the null hypothesis (for RTs, BF_01_ = 5.0, for errors, BF_01_ = 5.9).

#### SOA and Response-Repetition Effects on RTs and Error Rates

The results of the mixed models on RTs and error probability are reported in Table 3. For brevity, we only discuss the significant results of SOA and response relation.

**Table 3:** Mixed Models on Reaction Times and Errors of Experiment 3.


FIXED EFFECT	REACTION TIMES			ERROR			
	
	ESTIMATE	*t* value	*p*	ESTIMATE	*z* value	*p*	ODD RATIO

(Intercept)	699.921	30.37	<.001	–2.955	–33.724	<.001	0.052

Task Rep.	37.655	14.279	<.001	0.202	5.007	<.001	1.224

Context Rep.	2.693	1.08	0.28	–0.044	–1.155	0.248	0.957

SOA	–0.014	–1.37	0.171	0.009	0.184	0.854	1.009

Response Rep.	11.459	4.934	<.001	–0.058	–1.464	0.143	0.944

Task Rep.:Context Rep.	–1.363	–0.543	0.587	0.009	0.24	0.811	1.009

Task Rep.:SOA	0.108	10.679	<.001	0.005	0.108	0.914	1.005

Context Rep.:SOA	0.012	1.193	0.233	–0.08	–1.79	0.073	0.923

Context Rep.:Response Rep.	–0.54	–0.233	0.816	0.027	0.689	0.491	1.027

SOA:Response Rep.	–0.007	–1.076	0.282	–0.062	–1.815	0.07	0.94

Task Rep.:Context Rep.:SOA	0.019	1.961	0.05	–0.05	–1.054	0.292	0.951

Context Rep.:SOA:Response Rep.	–0.01	–1.453	0.146	0.045	1.309	0.191	1.046


*Note*. Rep. = Repetition. The variables are sum-coded such that Switch = 1 and Repetition = –1. SOA values were standardized. The model formula was Task_rep*Context_rep*SOA + Response_rep*Context_rep*SOA + (1|pp).

In the results of the linear mixed model on RTs, we found no main effect of SOA, unlike Experiment 1. However, we replicated the interaction between task relation and SOA, indicating larger task-repetition benefits with longer SOA. The three-way interaction, additionally including context relation, showed a p-value exactly at the significance threshold: the increase in task-repetition benefits with longer SOA was more pronounced in context repetitions than switches. As in Experiment 2, we found a significant effect of response relation, indicating shorter RTs in response repetitions (610 ms) than switches (690 ms). However, response relation did not interact with context relation. The generalised mixed model predicting the probability of making an error did not show any effects of response relation or SOA.

#### Correlations between Repetition Benefits and Switching Behaviour

As in the previous experiments, the task-repetition rate was positively correlated with the task-repetition benefits, ρ = 0.39, *t*(46) = 2.90, *p* = .006, indicating that participants with higher repetition benefits on average were also more willing to repeat the task. Different from the previous experiments, the correlation between repetition benefits and SOA was not significant, ρ = 0.24, *t*(46) = 1.66, *p* = .103. Finally, as in Experiments 1 and 2, participants’ average SOA in switch trials (200 ms) was significantly larger than their average repetition benefit (135 ms), *t*(47) = 3.73, *p* < .001, *d_z_* = 0.54, indicating an excessive willingness to wait compared to their individual average repetition benefits.

### Discussion

Despite the different context operationalisation, the results of Experiment 3 resemble those of Experiments 1 and 2. Although we managed to increase context saliency—as suggested by the faster responses in context repetitions than switches—context repetitions did not increase task-repetition rate. However, the Bayesian test only provided inconclusive support for the absence of an effect, unlike Experiments 1 and 2, where the evidence was substantial. Once again, context repetition did not increase task-repetition benefits, supported by substantial evidence for the null.

As before, longer SOA augmented task-repetition benefits. In Experiment 3, this increase was larger in context repetitions than switches. The *p*-value of this result is exactly at the significant threshold, but if confirmed in future studies, it would be consistent with the notion that repeating the context retrieves the N–1 task set. Specifically, it might indicate that the longer the SOA, the stronger the retrieval of the task set triggered by the repeating context. Context repetition did not increase response-repetition benefits either, as we observed in Experiment 1.

Experiment 3 was the only one where response repetitions did not show a higher error probability than response switches. In Experiment 3, we ensured the probability that both stimulus categories repeated (e.g., two vowels and two even numbers in two subsequent trials) was the same as the probability that neither repeated (e.g., a vowel followed by a consonant and an even number followed by an odd number). This means that, given that participants repeated the task, they had the same number of trials where performing a response repetition was correct versus wrong. In Experiments 1 and 2, instead, the probability that neither category repeated was approximately 1.5 times higher than that both repeated. Hence, the fact that response repetitions were not more error-prone than switches might be due to some random guesses resulting in correct responses in Experiment 3.

Larger task-repetition benefits corresponded to higher task-repetition rates, but, unlike Experiments 1 and 2, larger repetition benefits did not correspond to longer waiting SOA. This might constitute indirect evidence that the context as operationalized in Experiment 3 perturbed task choice more than the context of Experiments 1 and 2, reducing behaviour optimization. Finally, the average switch SOA was larger than the average repetition benefit, consistent with the previous experiments.

## General Discussion

In this study, we tested whether repeating a task-irrelevant context affected task repetitions in a voluntary task-switching paradigm (VTS). We used an adaptive VTS procedure (Mittelstädt, Miller, et al., 2018) that yields a sufficient number of switch trials, avoiding instructions that constrain participants’ choice. Specifically, we examined whether repeating the context would increase task-repetition rate and task-repetition benefits. We derived this hypothesis from a theoretical account postulating feature binding and episodic retrieval in task switching (e.g., Altmann, 2011; Frings et al., 2020). Accordingly, we assumed that the task set and the context might get bound in each trial and that a context repetition would retrieve the N–1 task set, increasing its availability and therefore (i) the probability it was repeated, and (ii) the benefits of repeating it compared to switching it. However, we did not find support for these hypotheses across three experiments, and the Bayes Factors generally provided substantial evidence in favour of the absence of these effects. In Experiment 3, we increased context saliency to augment the probability of observing an effect thereof. Although we finally observed a benefit for context repetitions compared to switches, this did not interact with task-repetition benefits or task-repetition rates. Taken together, these null results challenge the assumption that binding and retrieval mechanisms operate pervasively. Note that the lack of binding effects might reflect a lack of binding in a given trial, or a lack of retrieval in the following trial. By keeping these possibilities in mind, we discuss two non-mutually exclusive reasons why the present setup represents a boundary condition for binding and retrieval effects: the task-irrelevance of the context, and the free-choice component.

The notion that repeating a task-irrelevant context does not bias task repetition rate is consistent with earlier VTS studies described in the introduction (Arrington & Logan, 2005; Jurczyk, Fröber, et al., 2021, but see Demanet et al., 2010). However, based on earlier *forced*-choice task-switching studies (Benini et al., 2022, 2023, 2024; Dreisbach & Wenke, 2011; Koch, Frings, et al., 2018; Schiltenwolf et al., 2024), we expected context binding effects even for task-irrelevant contexts. These studies indeed showed that repeating a task-irrelevant context modulated task-repetition benefits (Schiltenwolf et al., 2024) and increased response-repetition benefits in task repetitions (Benini et al., 2022, 2023, 2024; Koch, Frings, et al., 2018). These results are also consistent with the context being bound with and retrieving the *response*. We hypothesize that this could also be the case in the present VTS experiments, where the context appeared before the stimuli and remained on the screen during response execution. In fact, we examined whether repeating the context would improve performance in response repetitions, but this was only the case in Experiment 1. Overall, the present study provides little support for context-response binding in VTS, and provides evidence against the task set and the context getting bound in each trial, or the repeating context retrieving the N–1 task (or both).

In this study, we focused on bindings formed on the fly and lasting a few seconds. A different question would be if the entire previous trial history influences task choice. Specifically, as it was suggested to us during the review process, participants might associate a given task with a given context, so that the occurrence of that context would prompt the selection of the associated task. We explored this possibility post-hoc and, for each trial, we calculated which task was most frequently paired with any context up to that trial. We then assessed whether the chosen task accords with the thus far most frequent task-context pairing. Specifically, we tested whether the probability of choosing the most frequent context-task pairing was above chance. This was in fact the case in Experiment 1 and 2 (Exp 1: 53.8%, *t*(45) = 1.89, *p* = .032, Exp 2: 55.7%, *t*(46) = 2.57, *p* = .006), but not in Experiment 3: *t*(47) = –0.46, *p* = .675). This provides some initial indications that participants might form longer-term task-context associations so that the presence of a given context can drive task choice. These results accord with earlier free-choice studies that investigated whether the choice of switching is affected by presenting a target that was instead systematically associated with a task *transition* (switch versus repetition) during an early cued task-switching training phase. Re-encountering a stimulus in a VTS test phase increased switch rate if this stimulus was associated with a task switch compared to stimuli associated with a repetition (Chiu et al., 2020). However, Mendl and colleagues (Mendl et al., 2024) failed to obtain this result when they associated a given task transition probability with a task-*irrelevant* stimulus (i.e., the cue colour) that resembles the context of the present experiments. This suggests that a stimulus can influence task choice via retrieval of an associated task *transition*, but only if this stimulus is task relevant (i.e., it is the target) and not when it is task irrelevant (i.e., it is a context).

The second explanation for why context transition did not influence task repetition rates is that short-term binding and retrieval phenomena do not affect voluntary task selection. Task selection modes might differ in forced-choice task switching compared to VTS. In forced-choice task switching, a cue instructs which task to perform in each trial. As a consequence, in cued task-switching, task set activation might be more influenced by external stimuli as compared to VTS. This is indeed the reason why the VTS paradigm was developed in the first place (e.g., Demanet & Liefooghe, 2014). Note, however, that Pfister and colleagues (Pfister et al., 2010) found evidence for binding effects influencing free-choice responses. However, in the present study, participants needed to select a task set, not a response, as in Pfister et al.’s study. Also, the binding effects of Pfister et al.’s study concerned a response and its perceptual effect, as opposed to an irrelevant context. Taken together, these differences might determine the lack of context repetition effects in the present VTS study: Even if the context is bound to the task set in each trial and retrieves it when the context repeats in the next trial, this retrieval-triggered activation might not suffice to bias task choice. This might be even more the case when using an adaptive procedure that stimulates behaviour optimisation (i.e., deciding between repeating or switching based on the SOA and one’s own repetition benefits). With this setup, the choice of repeating versus switching the task requires additional decision-making processes that take into account the current SOA and one’s own switching performance (i.e., how costly it is for each individual to switch). Once a task set is selected and activated via this decision-making process, the further activation of the chosen task set or of the competing task set caused by context repetition might be irrelevant. Arguably, if it was this specific adaptive procedure that annihilates the effect of context-triggered retrieval of the task set, one would expect such an effect to emerge at least at short SOAs: such a short delay between the stimuli reduces the need and the time to engage in decision-making processes. Note that, however, since the SOA in each trial was perfectly predictable, participants might decide whether to switch or repeat the task even *before* the new trial started, for example, deciding to exploit the short SOA trial to perform an easier task repetition. In this case, task choice would be even more dependent on top-down processes, limiting the impact of contextual bottom-up processes in this setup. In line with this reasoning, Orr and Weismann (2011) indeed showed that bottom-up task set availability especially biases task choice when less top-down control is available.

Taken together, we suggest that the combination of context irrelevance and of the VTS setting is responsible for the lack of context binding effects in the present study: In line with previous literature, bindings involving a task irrelevant context might be weaker (Memelink & Hommel, 2013) due to less attention devoted to the context (Schmalbrock et al., 2023, but see Laub & Frings, 2024). In addition, a VTS procedure calls for enhanced top-down control over task set retrieval, annihilating context-triggered retrieval.

Naturally, the principle of this adaptive procedure is grounded in bottom-up influences on task choice, since it consists of rendering the stimulus affording a task switch more available than the stimulus affording a task repetition (Mittelstädt, Miller et al., 2018). Hence, one could argue that, rather than choosing the task in each trial, participants choose the “stimulus set”, namely, they bias their attention to a given (task-irrelevant) position (Arrington & Weaver, 2015). In the present setup, this possibility cannot be distinguished from choosing the “response set”, namely the categories (e.g., odd/even): Committing to certain categories is equivalent to committing to one of the two locations, and vice versa. Moreover, the stimulus affording a switch is visible during the SOA: this allows us to accumulate stimulus-triggered evidence for a given response (e.g., “odd”). Hence, the possibility that participants chose a response, rather than a task, might be even more warranted in this setup.

In VTS, the possibility that participants select the category to respond to—and end up executing a given task as a consequence, not as a cause—has been discussed (e.g., Mittelstädt, Miller, et al., 2018; Orr & Weissman, 2011, see also Forstmann et al., 2006) in opposition to the notion that participants need to first select a task to then execute it (Arrington & Logan, 2005; Vandierendonck et al., 2012). If task choice does not strictly precede task execution, a task-context binding might not show because participants’ behaviour would reflect the choice of the stimulus-set or response-set instead. In this case, binding effects only emerge if our irrelevant box/image (i.e., the context) becomes bound with the stimulus location or with the response categories in trial N–1, instead of with the task set. Hence, repeating the context retrieves and activates the N–1 location and/or category in trial N. This would increase, in context repetitions, the probability of responding to the same stimulus location or with the same response set, which corresponds to a larger probability of repeating the task. Hence, a context-location or context-category binding would yield a result that is observationally equivalent to our (not confirmed) hypothesis, despite not reflecting a task-set context binding. Therefore, the null result in our three experiments also speaks against the possibility of context-location or context-category binding and retrieval processes.

Across the three experiments, we consistently observed that longer SOAs sped up task repetitions and slowed down task switches. Note that this result is not related to our hypotheses involving context retrieval effects. In task repetitions, the RT reflects the time from the onset of the repetition stimulus to the response thereto, so that it does not include the SOA. Hence, participants might have used the SOA to prepare for a task repetition. Such preparation reflects the activation of the task set representation, but certainly not of a response, since the repetition stimulus was not visible during the SOA. The effect of manipulating the interval between a response and the next trial, or between the cue and the stimulus, has been extensively examined in forced-choice task switching (e.g., Koch, 2003; Mayr & Kliegl, 2003; Monsell & Mizon, 2006; see also Monsell, 2017). For the present discussion, the closest parallel can be drawn with task-switching studies using predictable task sequences, where the whole interval between a response and the next stimulus can be used to prepare for the upcoming, predictable task (e.g., Rogers & Monsell, 1995). When participants can predict the interval length and know the upcoming task, typically repetition benefits shrink (Rogers & Monsell, 1995; see also Koch, 2008, for a discussion) and, sometimes, performance improves overall (Koch, 2005). This reduction of repetition benefits was also observed in VTS (e.g., Demanet & Liefooghe, 2014; Liefooghe et al., 2009). In the present study, with a longer time before the onset of the repetition stimulus, task-repetition benefits increased. This would be consistent with the interpretation that participants used the SOA to prepare for task repetitions: If they eventually decided to switch tasks—potentially even before the whole SOA elapsed—the repetition task might be more strongly prepared and thus harder to overcome, yielding longer RTs.

A second explanation for this effect of SOA on task-repetition benefits comes from a longer SOA being confounded with a longer run of the same task (i.e., more consecutive task repetitions). In cued task switching (but not in task-switching with predictable sequences), it was shown that longer runs of repetitions of the same task improve performance compared to repetitions belonging to shorter runs (Monsell et al., 2003). Hence, what looks like an effect of longer SOA might in fact be an effect of consecutive task repetitions. A third explanation is that participants might decide to switch tasks only after they could see the repetition stimulus, which would naturally add the SOA duration to switch RTs, again yielding larger task-repetition benefits with longer SOAs.

## Conclusion

Bottom-up influences play a well-established role in task choice and task availability in VTS. However, our study provides convincing evidence for the absence of bottom-up effects driven by a task-irrelevant context. We operationalized the context as a coloured box or a picture that could repeat or switch in subsequent trials. This operationalization yielded results consistent with task set-context binding and retrieval mechanisms in previous forced-choice task-switching experiments. Hence, we expected a context repetition to increase task-repetition rates compared to a context change. In three VTS experiments, we found no evidence to support this prediction, which challenges the assumption that binding and retrieval mechanisms also concern task-irrelevant contextual features in VTS. Another possibility is that task and context were only weakly associated in our design. Finally, the adaptive VTS procedure that we used may have encouraged stronger top-down control on task choice, limiting the impact of repeating or switching the irrelevant context.

## Data Availability

The data and the code to reproduce the analyses are available at: https://doi.org/10.23668/psycharchives.21595.

## Footnotes

For completeness, other studies showed that the availability of each task set representation is additionally influenced by the cognitive control state participants are in (Dreisbach & Fröber, 2019). Cognitive control state refers to a configuration of the cognitive control system (i.e., how attention is deployed on task relevant versus irrelevant stimuli, response biases, which information is maintained, etc.) that depends on the goal at end (Botvinick et al., 2001). For example, a setting of frequent switching might shift the control state from a stable to a flexible control state that reduces switch costs (Siqi-Liu & Egner, 2020). Our study is not concerned with manipulation of control states, hence reviewing this literature is beyond the scope of this work, but, in a nutshell, repetition rates were shown to decrease with a flexible—as opposed to stable—cognitive control state (Fröber et al., 2022; Fröber & Dreisbach, 2017, see also Dreisbach & Mendl, 2024 for a review).

These analyses were preregistered for Experiments 2 and 3, but for Experiment 1, we preregistered an ANOVA on task-repetition rate as a function of context relation and SOA, and ANOVAs on RTs and error rates again as a function of context relation and SOA.
